# Trivariate mover‐stayer counting process models for investigating joint damage in psoriatic arthritis

**DOI:** 10.1002/sim.7074

**Published:** 2016-08-08

**Authors:** Sean Yiu, Brian D. M. Tom, Vernon T. Farewell

**Affiliations:** ^1^ MRC Biostatistics UnitCambridgeCB2 0SRU.K.

**Keywords:** intermittent observations, longitudinal count data, mover‐stayer model, psoriatic arthritis, random effects

## Abstract

In psoriatic arthritis, many patients do not develop permanent joint damage even after a prolonged follow‐up. This has led several authors to consider the possibility of a subpopulation of stayers (those who do not have the propensity to experience the event of interest), as opposed to assuming the entire population consist of movers (those who have the propensity to experience the event of interest). In addition, it is recognised that the damaged joints process may act very differently across different joint areas, particularly the hands, feet and large joints. From a clinical perspective, interest lies in identifying possible relationships between the damaged joints processes in these joint areas for the movers and estimating the proportion of stayers in these joint areas, if they exist. For this purpose, this paper proposes a novel trivariate mover‐stayer model consisting of mover‐stayer truncated negative binomial margins, and patient‐level dynamic covariates and random effects in the models for the movers and stayers, respectively. The model is then extended to have a two‐level mover‐stayer structure for its margins so that the nature of the stayer property can be investigated. A particularly attractive feature of the proposed models is that only an optimisation routine is required in their model fitting procedures. © 2016 The Authors. Statistics in Medicine Published by John Wiley & Sons Ltd.

## 1 Introduction

Psoriatic arthritis (PsA) is an inflammatory arthritis associated with the skin condition psoriasis. A basic measure of disease progression in PsA is the attained number of permanently damaged joints. Since its inception in 1978, the University of Toronto PsA clinic has established the largest and most comprehensively studied cohort of PsA patients in the world. A notable feature of this cohort is the large number of patients whose disease process does not progress to permanent joint damage, even after a prolonged follow‐up. Thus, when characterising the rate of accumulating damaged joints, there is generally a greater amount of zeros than is predicted from a standard count distribution. Several authors have considered mover‐stayer damaged joints counting process models in order to understand the damage process at the total joint level (all joints, Aguirre‐Hernández and Farewell 1) or with regard to the joints in the hands (Cook *et al.*, 2, Solis‐Trapala and Farewell 3, and O'Keeffe *et al.*, 4). These models estimate the proportion of stayers (those who do not have the propensity to experience the event of interest), through a binary component, and describe the counting process of the movers (those who have the propensity to experience the event of interest) with regard to the occurrence of clinical joint damage over time. Sutradhar and Cook 5 provided an extension by developing a bivariate mover‐stayer damaged joints counting process model for the situation where two damaged joints counting processes are of interest. A multinomial distribution was specified for the joint binary component, whilst a random effect was used to correlate the rates for the movers. Their model was clinically motivated by distinguishing between clinical and radiological damage at the total joint level. Note that these works have focused on two or less damaged joints counting processes with one stayer population being associated with each process.

In the present setting, the clinical aims are to identify possible relationships between accumulating damaged joints in the hands, feet and large joints for the movers, which may be asymmetric, and to estimate the stayer proportions in each joint area. Hence, three processes are now of interest. This would provide further understanding of the damage joints counting process in each joint area (hands, feet and large joints), which are expected to be different, and contribute new understanding of the possibly global nature of damage. In addition, it would also be of interest to investigate the nature of the stayer property, particularly to distinguish between patients who are inherent stayers (true stayers) or attain the stayer property through management/treatment strategies employed by the clinic (clinic‐induced stayers). These clinical considerations motivate the need for the development of new methodology that allows the relationship between three damaged joints counting processes to be investigated whilst simultaneously allowing for the possibility that there could be two stayer populations associated with each process.

In the count data setting, mover‐stayer models are referred to as zero‐inflated models, and these are commonly used when count data sets display a large number of zeros. The zero‐inflated Poisson (ZIP) model was first considered by Lambert 6. Böhning 7 and Ridout *et al.*, 8 provide a comprehensive review of this methodology, whilst Böhning *et al.*, 9 demonstrate various applications to the public health and social science settings. When the study design also results in clustered observations, a popular modification is to incorporate cluster‐specific random effects. These models can however be computationally intensive, primarily because of the required integration over the random effects. Hall 10 used the expectation‐maximization (EM) algorithm to fit a ZIP model which included a random effect in the Poisson and not the binary component of the model. The ZIP model of Hur *et al.*, 11 contained distinct random effects in both components of the model, although these authors chose to use numerical quadrature and a Newton‐Ralphson solution to simultaneously estimate the parameters of their model. They state the advantages of this routine over the EM algorithm are easily obtainable standard errors and a quicker convergence rate. Lee *et al.*, 12 and recently, Morgan *et al.*, 13 extended these models to allow for a further level of clustering. This was performed by incorporating two separate random effects into each component. The former utilised the EM algorithm, whilst the latter took a Bayesian approach in the model fitting procedure. We note that marginal models have been proposed to handle clustering. These models have been constructed through generalised estimating equations that allow a working correlation matrix to be incorporated into the model fitting procedure, see Dobbie and Welsch 14 for an example.

Motivated by the aforementioned clinical considerations, we develop a novel trivariate model with mover‐stayer truncated negative binomial margins, as a flexible alternative to ZIP models, and incorporate patient‐level dynamic covariates and random effects in the models for the movers and stayers, respectively. The dynamic covariates allow asymmetric relationships to be identified, whilst the random effects provide information across processes to estimate the stayer proportions. We then extend this model to have a two‐level mover‐stayer structure for its margins so that inherent and clinic‐induced stayers can be investigated for each marginal process. Patient‐level random effects are again incorporated in each stayer/binary component. In contrast to the literature where the logit link function and the distributional assumption of normal random effects are usually specified for the binary component, we consider the complementary log‐log link function, and this allows a closed form marginal likelihood to be obtained if the distributional assumption of the random effects is chosen to have a closed form Laplace transform. Thus, computationally intensive techniques such as numerical integration, EM algorithm and MCMC are not required in the model fitting procedure. This is particularly useful for the implementation of the extended model because it contains two patient‐level random effects that must be integrated out for each patient's likelihood contribution.

The next section introduces the PsA data on which this analysis is undertaken.

## 2 Psoriatic arthritis data

**Figure 1:**
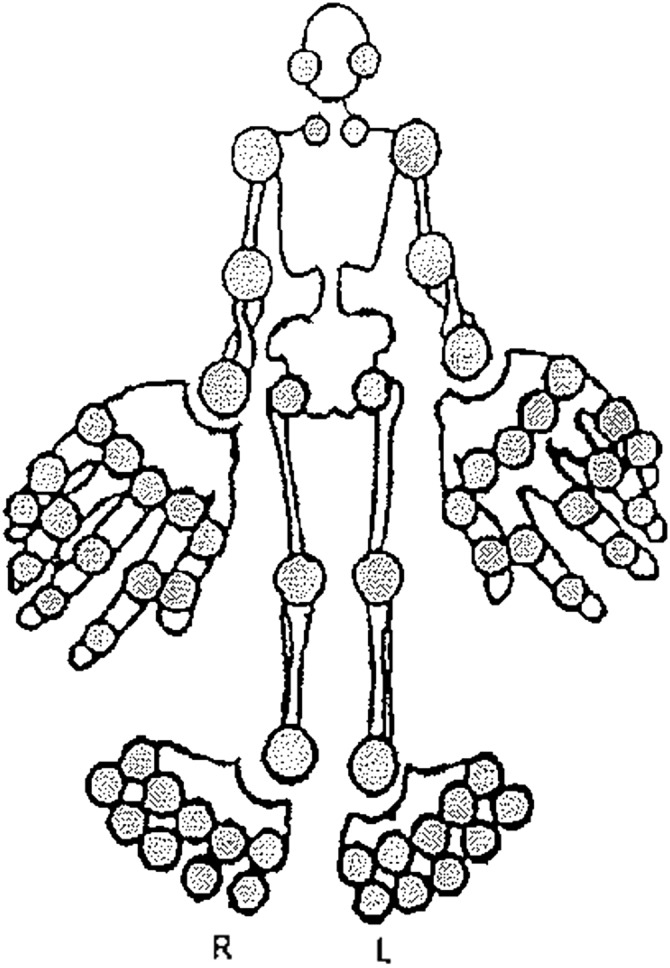
The joints considered in these analyses are the right and left jaw, sterno‐clavicular, shoulder, elbow, wrist, hip, hand, knee, ankle and foot. Joints are listed from top to bottom as they are displayed in the figure.

This analysis focuses on a longitudinal data set containing the follow‐up history of 1194 patients from the University of Toronto PsA clinic. At each clinic visit, which were scheduled 6–12 months apart, various clinical measurements including the active (swollen and/or painful) and damaged joints counts were obtained. In particular, joint counts are recorded at the individual joint level, therefore allowing the damaged joints counting processes to be examined for the 28 hand joints (14 in each hand), 20 foot joints (10 in each foot) and 16 large joints. The large joints consist of the left and right jaw, sterno‐clavicular, shoulder, elbow, wrist, hip, knee and ankle. Figure 1 displays the location of these joints.

Of the 1194 patients, 997 had more than one clinic visit. At clinic entry, the mean age was 37 years, with standard deviation of 13 years and 4 months, whilst the mean disease duration was 7 years with standard deviation of 8 years and 3 months. The mean follow‐up time for those who had more than a single clinic visit was 9 years and 5 months, with interquartile range of 11 years and 1 month, and their mean and median inter‐visit times were 10 and 6 months, with standard deviation of 1 year and 2 months.

**Figure 2:**
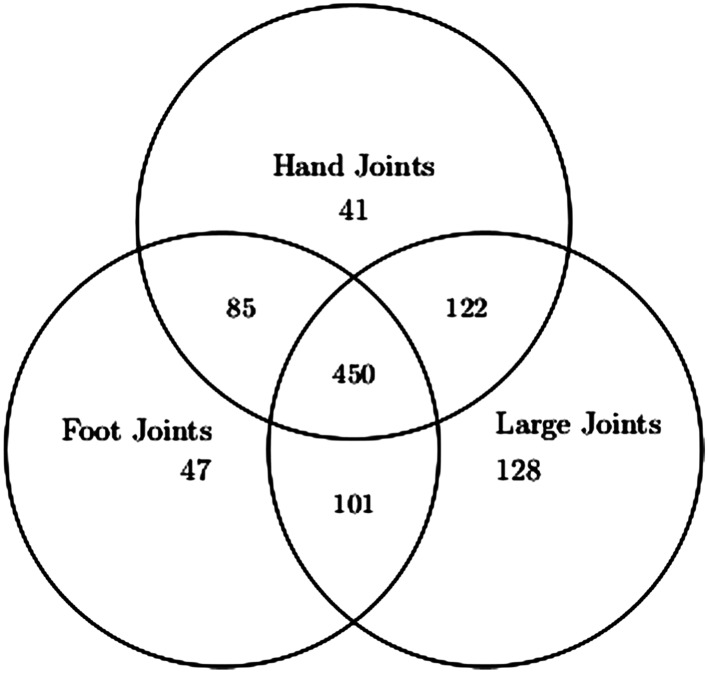
Venn diagram containing the frequencies of the 1194 psoriatic arthritis patients who remained damage free in the various combinations of joint areas.

As mentioned, a notable feature of this data set is the large proportion of patients who did not develop damaged joints in these joint areas. There were 698 (58*%*), 683 (57*%*) and 801 (67*%*) patients who remained damage free in the hands, feet and large joints, respectively. Figure 2 provides more details by displaying the frequencies of patients who remained damage free in the different combination of joint areas. For example, of the 801 patients who remained damage free in the large joint locations, 450 patients did not develop any damage in the hand or foot joints, 122 did not develop any damage in the hand joints but did so in the foot joints, 101 did not develop any damage in the foot joints but did so in the hand joints, and 128 developed damage in both the hand and foot joints.

In the next section, a trivariate mover‐stayer damaged joints counting process model is developed for the hands, feet and the large joints.

## 3 Model for trivariate mover‐stayer damaged joints counting processes

### 3.1 Model

Let $N_{\mathrm{ij}}^{k}$ be random variables representing the total number of damaged joints that patient *i* has accumulated in joint area *k* up to the time of the *j*th clinic visit *t*_*ij*_. Then, $D_{\mathrm{ij}}^{k}=N_{\mathrm{ij}+1}^{k}-N_{\mathrm{ij}}^{k}$ represents the number of damaged joints developed in joint area *k* between *t*_*ij*_ and *t*_*ij* + 1_ with *j* = 0,…,*m*_*i*_−1 and *k* = *h*, *f* and *l* denoting the hands, feet and large joints, respectively. Here, *m*_*i*_ represents the number of clinic visits for patient *i*. Suppose that $D_{\mathrm{ij}}^{k}$ is independent of $\left\{D_{\mathrm{ir}}^{h},D_{\mathrm{ir}}^{f},D_{\mathrm{ir}}^{l},r=0,\ldots j-1\right\}$ conditional on the inclusion of the dynamic covariates $N_{\mathrm{ij}}^{h}$, $N_{\mathrm{ij}}^{f}$ and $N_{\mathrm{ij}}^{l}$. A regression model can be specified by allowing $D_{\mathrm{ij}}^{k}$ to have a negative binomial distribution with mean $\Lambda_{\mathrm{ij}}^{k}$ and dispersion parameter $\theta^{k}\geq 0$ that is truncated to account for the number of joints that have the propensity to become damaged;

$$P\;\left(D_{\mathrm{ij}}^{k}=d_{\mathrm{ij}}^{k}|{\left\{N_{\mathrm{ij}}^{k}=n_{\mathrm{ij}}^{k}\right\}}_{k=h,f,l}\right)=\frac{f_{\mathrm{nb}}\left(d_{\mathrm{ij}}^{k}|\Lambda_{\mathrm{ij}}^{k},\theta^{k}\right)}{\overset{T^{k}-n_{\mathrm{ij}}^{k}}{\underset{r=0}{\sum }}f_{\mathrm{nb}}\left(r|\Lambda_{\mathrm{ij}}^{k},\theta^{k}\right)},$$

where *T* ^*k*^=28, 20 and 16 for *k* = *h*, *f* and *l*, respectively. Here, *T* ^*k*^ specifies the number of joints in the *k*th joint area,

$$f_{\mathrm{nb}}(r|\Lambda ,\theta )=\frac{\Gamma \left(r+\frac{1}{\theta }\right)}{\Gamma \left(\frac{1}{\theta }\right)r!}{\left(\frac{\theta \Lambda }{1+\theta \Lambda }\right)}^{r}{\left(\frac{1}{1+\theta \Lambda }\right)}^{\frac{1}{\theta }}$$

and

$$\Lambda_{\mathrm{ij}}^{k}=\left(t_{\mathrm{ij}+1}-t_{\mathrm{ij}}\right)\lambda_{0}^{k}\exp\;\left(\overset{k}{\underset{1}{\beta }}\overset{k}{\underset{i1}{z}}(t_{\mathrm{ij}})+\ldots +\beta_{p-3}^{k}z_{\mathrm{ip}-3}^{k}(t_{\mathrm{ij}})+\beta_{p-2}^{k}n_{\mathrm{ij}}^{h}+\beta_{p-1}^{k}n_{\mathrm{ij}}^{f}+\beta_{p}^{k}n_{\mathrm{ij}}^{l}\right),$$

where $\lambda_{0}^{k}$ is a constant baseline intensity, $\left(z_{i1}^{k}(t_{\mathrm{ij}}),\ldots ,z_{\mathrm{ip}-3}^{k}(t_{\mathrm{ij}})\right)$ are covariates evaluated at *t*_*ij*_ and $\left(\beta_{1}^{k},\ldots ,\beta_{p}^{k}\right)$ are regression coefficients.

To reflect the possibility that a subpopulation of stayers may exist, a partially observable binary variable $C_{i1}^{k}$, where $C_{i1}^{k}=0$ if patient *i* is a stayer in joint area *k* and $C_{i1}^{k}=1$ otherwise, can be incorporated into the model. These variables are partially observable because they are known for a particular joint area of a patient if damage has occurred, that is $C_{i1}^{k}=1$, otherwise, they are unknown. The probability that patient *i* is a stayer in joint area *k* conditional on a patient‐level random effect *U*_*i*_=*u*_*i*_ can then be estimated as

$$\pi_{i1}^{k}:=\pi_{i1}^{k}\left(\alpha_{1}^{k}|z_{i⋆}^{k},u_{i}\right)=P\;\left(C_{i1}^{k}=0|z_{i⋆}^{k},u_{i}\right)=1-\exp\;\left(-u_{i}\exp\;\left(\overset{k^{\prime }}{\underset{1}{\alpha }}z_{i⋆}^{k}\right)\right),$$

where $z_{i⋆}^{k}$ and $\alpha_{1}^{k}$ are column vectors of time‐invariant covariates and regression coefficients, respectively. The random effect *U*_*i*_, which is incorporated at the patient‐level, reflects the characteristic that the stayer probabilities across locations are likely to be more similar within patients.

Furthermore, if the stayer property is inherent (immune to damage before and after clinic entry), the information obtained between arthritis onset and clinic entry will contribute towards estimation of the stayer proportions, and thus, this information must be accounted for in the analysis. Let $D_{i0^{*}}^{k}$ be the number of damaged joints patient *i* developed in area *k* in this period. A model for $D_{i0^{*}}^{k}$ given that no damaged joints at arthritis onset is possible, that is $N_{i0^{*}}^{k}=0$, can again be specified as a negative binomial distribution with mean $\Lambda_{i0}^{k}=\left(t_{i0}-t_{i0^{*}}\right)\lambda_{0}^{k0}$ and dispersion parameter *θ* ^*k*0^ and having again the relevant truncation. Here, $\lambda_{0}^{k0}$ is a constant baseline intensity, and $t_{i0^{*}}$ is the time of arthritis onset for patient *i*. That is

$$P\;\left(D_{i0^{*}}^{k}=n_{i0}^{k}|N_{i0^{*}}^{k}=0\right)=\frac{f_{\mathrm{nb}}\left(n_{i0}^{k}|\Lambda_{0}^{k},\theta^{k_{0}}\right)}{\overset{T^{k}}{\underset{r=0}{\sum }}f_{\mathrm{nb}}\left(r|\Lambda_{0}^{k},\theta^{k_{0}}\right)}.$$

Note that inferences from these models are not of primary interest; their use is to provide information towards the estimation of the stayer proportions.

Under this formulation, the conditional likelihood contribution from patient *i* in joint area *k* (given the patient‐level random effect *U*_*i*_=*u*_*i*_ and the dynamic covariates, the total number of damaged joints in each joint area ${\left\{N_{\mathrm{ij}}^{k}=n_{\mathrm{ij}}^{k}\right\}}_{k=h,f,l}$), is firstly, if no damaged joints are observed;

$$\begin{array}{c} P\;\left(N_{i0}^{k}=0,\ldots ,N_{im_{i}}^{k}=0|N_{i0^{*}}^{k}=0;u_{i}\right)\\ =P\;\left(D_{i0^{*}}^{k}=0,\ldots ,D_{im_{i}-1}^{k}=0|u_{i}\right)\\ =P\;\left(D_{i0^{*}}^{k}=0,\ldots ,D_{im_{i}-1}^{k}=0|C_{i1}^{k}=0\right)P\;\left(C_{i1}^{k}=0|u_{i}\right)\\ \;+P\;\left(D_{i0^{*}}^{k}=0,\ldots ,D_{im_{i}-1}^{k}=0|C_{i1}^{k}=1\right)P\;\left(C_{i1}^{k}=1|u_{i}\right)\\ =\pi_{i1}^{k}+\left(1-\pi_{i1}^{k}\right)\overset{m_{i}-1}{\underset{j=0}{\prod }}P\;\left(D_{\mathrm{ij}}^{k}=0|{\left\{N_{\mathrm{ij}}^{k}=n_{\mathrm{ij}}^{k}\right\}}_{k=h,f,l}\right)P(D_{i0^{*}}^{k}=0|N_{i0^{*}}^{k}=0), \end{array}$$

and secondly, if $n_{i0}^{k},\ldots ,n_{im_{i}}^{k}$ damaged joints are observed at $t_{i0},\ldots ,t_{im_{i}}$ and $n_{im_{i}}\neq 0$;

$$\begin{array}{c} \;P\;\left(N_{i0}^{k}=n_{i0}^{k},\ldots ,N_{im_{i}}^{k}=n_{im_{i}}^{k}|N_{i0^{*}}^{k}=0;u_{i}\right)\\ =P\;\left(D_{i0^{*}}^{k}=n_{i0}^{k},D_{i0}^{k}=d_{i0}^{k},\ldots ,D_{im_{i}-1}^{k}=d_{im_{i}-1}^{k}|u_{i}\right)\\ =P\;\left(D_{i0^{*}}^{k}=n_{i0}^{k},D_{i0}^{k}=d_{i0}^{k},\ldots ,D_{im_{i}-1}^{k}=d_{im_{i}-1}^{k}|C_{i1}^{k}=0\right)P\;\left(C_{i1}^{k}=0|u_{i}\right)\\ \;+P\;\left(D_{i0^{*}}^{k}=n_{i0}^{k},D_{i0}^{k}=d_{i0}^{k},\ldots ,D_{im_{i}-1}^{k}=d_{im_{i}-1}^{k}|C_{i1}^{k}=1\right)P\;\left(C_{i1}^{k}=1|u_{i}\right)\\ =\left(1-\pi_{i1}^{k}\right)\overset{m_{i}-1}{\underset{j=0}{\prod }}P\;\left(D_{\mathrm{ij}}^{k}=d_{\mathrm{ij}}^{k}|{\left\{N_{\mathrm{ij}}^{k}=n_{\mathrm{ij}}^{k}\right\}}_{k=h,f,l}\right)P\;\left(D_{i0^{*}}^{k}=n_{i0}^{k}|N_{i0^{*}}^{k}=0\right), \end{array}$$

where $d_{\mathrm{ij}}^{k}=n_{\mathrm{ij}+1}^{k}-n_{\mathrm{ij}}^{k}$ for *j* = 0,…,*m*_*i*_−1. The likelihood contribution *L*_*i*_(Θ) (where all unknown parameters are contained in the vector Θ) from patient *i* can then be derived by assuming that conditional on the random effect *U*_*i*_ and dynamic covariates $N_{i}^{h}$, $N_{i}^{f}$ and $N_{i}^{l}$, the mover‐stayer damaged joints counting processes within an individual are independent. Hence,

$$L_{i}(\Theta )=\overset{\infty }{\underset{0}{\int }}L_{i}^{h}(\Theta |u_{i})L_{i}^{f}(\Theta |u_{i})L_{i}^{l}(\Theta |u_{i})g(u_{i}|\gamma )\;du_{i}$$

where

$$\begin{array}{cc} L_{i}^{k}(\Theta |u_{i}) & ={\left\{1-\exp\;\left(-u_{i}\omega_{i}^{k}\right)+\exp\;\left(-u_{i}\overset{k}{\underset{i}{\omega }}\right)\overset{k}{\underset{i}{P}}\right\}}^{1-c_{i*}^{k}}{\left\{\exp\;\left(-u_{i}\omega_{i}^{k}\right)\overset{k}{\underset{i}{P}}\right\}}^{c_{i*}^{k}}\\ P_{i}^{k} & :=\overset{m_{i-1}}{\underset{j=0}{\prod }}P\;\left(D_{\mathrm{ij}}^{k}=d_{\mathrm{ij}}^{k}|{\left\{N_{\mathrm{ij}}^{k}=n_{\mathrm{ij}}^{k}\right\}}_{k=h,f,l}\right)P\;\left(D_{i0^{*}}^{k}=n_{i0}^{k}|N_{i0^{*}}^{k}=0\right)\\ \omega_{i}^{k} & :=\exp\;\left(\alpha^{k^{\prime }}z_{i⋆}^{k}\right) \end{array}$$

for *k* = *h*, *f*, *l* and, $c_{i*}^{k}=0$ if $N_{im_{i}}^{k}=0$ and $c_{i*}^{k}=1$ otherwise. The conditional likelihood is then computed by taking the product of all conditional likelihood contributions from each patient. The next subsection provides details on how the integration over the random effects can be computed analytically.

### 3.2 Computing the likelihood analytically

Firstly, the conditional likelihood contribution from patient *i* in joint area *k* can be rearranged as

$$\begin{array}{c} \;\;{\left\{1-\exp\;\left(-u_{i}\omega_{i}^{k}\right)+\exp\;\left(-u_{i}\overset{k}{\underset{i}{\omega }}\right)\overset{k}{\underset{i}{P}}\right\}}^{1-c_{i*}^{k}}{\left\{\exp\;\left(-u_{i}\omega_{i}^{k}\right)\overset{k}{\underset{i}{P}}\right\}}^{c_{i*}^{k}}\\ ={\left\{1-\exp\;\left(-u_{i}\omega_{i}^{k}\right)\left(1-\overset{k}{\underset{i}{P}}\right)\right\}}^{1-c_{i*}^{k}}{\left\{\exp\;\left(-u_{i}\omega_{i}^{k}\right)\overset{k}{\underset{i}{P}}\right\}}^{c_{i*}^{k}}\\ =\overset{1-c_{i*}^{k}}{\underset{r_{k}=0}{\sum }}{\left(-1\right)}^{r_{k}}{\left\{P_{i}^{k}\right\}}^{c_{i*}^{k}}{\left\{1-P_{i}^{k}\right\}}^{r_{k}\left(1-c_{i*}^{k}\right)}\exp\;\left(-u_{i}\omega_{i}^{k}\left(c_{i*}^{k}+r_{k}\left(1-c_{i*}^{k}\right)\right)\right), \end{array}$$

where the form of the third line can be seen by comparing terms when $c_{i*}^{k}=1$ and 0, respectively. The conditional likelihood contribution from patient *i* is then

$$\begin{array}{cc} L_{i}(\Theta |u_{i}) & =\underset{k=h,f,l}{\prod }{\left\{1-\exp\;\left(-u_{i}\omega_{i}^{k}\right)+\exp\;\left(-u_{i}\overset{k}{\underset{i}{\omega }}\right)\overset{k}{\underset{i}{P}}\right\}}^{1-c_{i*}^{k}}{\left\{\exp\;\left(-u_{i}\omega_{i}^{k}\right)\overset{k}{\underset{i}{P}}\right\}}^{c_{i*}^{k}}\\ =\overset{1-c_{i*}^{h}}{\underset{r_{h}=0}{\sum }}\overset{1-c_{i*}^{f}}{\underset{r_{f}=0}{\sum }}\overset{1-c_{i*}^{l}}{\underset{r_{l}=0}{\sum }}{\left(-1\right)}^{r_{h}+r_{f}+r_{l}}\left[\underset{k=h,f,l}{\prod }{\left\{P_{i}^{k}\right\}}^{c_{i*}^{k}}{\left\{1-P_{i}^{k}\right\}}^{r_{k}\left(1-c_{i*}^{k}\right)}\right]\\ \;\times \exp\;\left(-u_{i}\underset{k=h,f,l}{\sum }\omega_{i}^{k}\left(c_{i*}^{k}+r_{k}\left(1-c_{i*}^{k}\right)\right)\right). \end{array}$$

From this parametrisation, it is evident that the marginal likelihood contribution from patient *i* can be computed analytically if the distributional choice for *U*_*i*_ has a closed form Laplace transform. In this paper, as suggested by Conaway 15, the gamma distribution with unit mean and variance *γ* is considered for *U*_*i*_. Its Laplace transform is given by

$$E(\exp(-su_{i}))={\left(\frac{1}{1+\mathrm{\gamma s}}\right)}^{\frac{1}{\gamma }}.$$

Under this distributional assumption, the marginal form for $\pi_{i1}^{k}$(integrating out *u*_*i*_) is then

$$\pi_{i1}^{k*}\left(\alpha_{1}^{k},\gamma |z_{i⋆}^{k}\right)=1-{\left(\frac{1}{1+\gamma \exp\;\left(\overset{k^{\prime }}{\underset{1}{\alpha }}z_{i⋆}^{k}\right)}\right)}^{\frac{1}{\gamma }}.$$

This function, which is similar to the one used by Pregibon 16, contains the logit link when *γ* = 1 and the complementary log‐log link as *γ*→0. It can therefore be used to validate the suitability of these particular link functions as well as to offer greater flexibility.

## 4 Application

The model described in Section 3, which will be denoted the full model, was fitted to the 1194 PsA patients described in Section 2. In addition to the dynamic covariates, the attained number of damaged joints in each location; the current number of active joints in the hands, feet and large joints were also considered as covariates in the truncated negative binomial components. In a preliminary analysis, gender, age and arthritis duration were demonstrated to be not statistically and significantly related to the risk of damaged joints (results not shown). For the binary components, intercept only models were considered so that the marginal stayer proportions could be more simply investigated.

Parameter estimation for this and all other subsequent models in this paper were achieved using the Broyden‐Fletcher‐Goldfarb‐Shanno (BFGS) 17 optimisation algorithm in the statistical package R 18. Other methods might be used and be more appropriate in some situations. The reported confidence intervals for all parameters are 95*%* Wald intervals obtained from evaluating and then inverting the observed information matrix at the maximum likelihood estimates.

Table 1 displays the results from the full model. Of primary interest is the relationship between damage progression in the hand and foot joints. The table indicates that the number of damaged hand joints is strongly and positively associated with damage progression in the foot joints, and that the reverse relationship (number of damaged foot joints on damage progression in the hand joints) is not statistically significant. This asymmetric relationship could be of clinical interest, especially as the strength of associations of the number of damaged hand joints (0.083) and active foot joints (0.1) when modelling damage progression in the foot joints are comparable in magnitude. Additionally, the attained number of damaged large joints demonstrates a significant positive association with damage foot joints progression, whilst little association with damage hand joints progression is seen. In contrast, the number of active joints only seems to have a local effect. The significant associations are only really seen within joint areas for these variables, except for the possibly counter intuitive negative association between the number of active foot joints and damage progression in the large joints. Because of the large joints category being composed of many different types of joints, it is not possible to draw any specific conclusions regarding this counter intuitive result. It is, however, interesting to note the positive association between the attained number of damaged hand joints and damage progression in the large joints. These results might prompt further investigation into the large joints category, and further highlights the importance of understanding the damaged hand joints process.

**Table 1:** Parameter estimates related to associations with damaged joint counts, and stayer probability estimates obtained from fitting the full model to 1194 psoriatic arthritis patients. The P(Stayer) estimates were calculated as $\pi_{1}^{k*}({\hat{\alpha }}_{1},\hat{\gamma })$ for *k* = *h*, *f* and *l*. Standard errors for these quantities were calculated using the delta method.

Damage progression	Hand joints	Foot joints	Large joints
Attained number of
damaged hand joints	0.099 (0.074, 0.12)	0.083 (0.042, 0.12)	0.033 (0.013, 0.053)
Current number of
active hand joints	0.11 (0.083, 0.14)	0.013 (−0.025, 0.051)	0.0031 (−0.027, 0.034)
Attained number of
damaged foot joints	−0.0045 (−0.023, 0.014)	0.036 (0.0015, 0.071)	0.012 (−0.011, 0.035)
Current number of
active foot joints	−0.0088 (−0.038, 0.021)	0.1 (0.058, 0.15)	−0.05 (−0.088, −0.013)
Attained number of
damaged large joints	−0.0076 (−0.064, 0.048)	0.11 (0.017, 0.21)	0.0068 (−0.056, 0.07)
Current number of
active large joints	0.019 (−0.044, 0.081)	0.039 (−0.044, 0.12)	0.31 (0.24, 0.38)
*λ*_0_	0.15 (0.13, 0.18)	0.22 (0.18, 0.27)	0.058 (0.046, 0.72)
$\lambda_{0}^{0}$	0.65 (0.48, 0.87)	0.54 (0.39, 0.76)	0.088 (0.07, 0.11)
*θ*	7.96 (7.07, 8.97)	13 (11.6, 14.5)	7.81 (6.29, 9.69)
*θ* ^0^	4.7 (3.91, 5.64)	6.5 (5.38, 7.85)	3.88 (2.87, 5.24)
P(Stayer)	0.37 (0.33, 0.41)	0.31 (0.26, 0.35)	0.29 (0.22, 0.35)
*γ*	3.9 (2.76, 5.51)		
Log‐likelihood	−13 532.86		

In all three joint areas, there is strong evidence of a subpopulation of stayers as the confidence interval for each of the relevant estimated probabilities are far from zero. The marginal stayer proportion estimates are 0.37 (0.33, 0.41), 0.31 (0.26, 0.35) and 0.29 (0.22, 0.35) for the hands, feet and large joints, respectively. Table 1 suggests, on average, the large joints experience the slowest damage progression rate. This may explain why the large joints category has the smallest estimated stayer proportion even though most patients remained damage free in this area. There is also evidence from Table 1 that a gamma distributed patient‐level random effect is required, and that the link function for the marginal binary components is neither the logit or complementary log‐log functional form $\left(\hat{\gamma }=3.9(2.76,5.51\right)$).

The inverse Gaussian distribution was also considered for *U*_*i*_, which is comparable to the normality assumption that is commonly employed in the literature. The resulting parameter estimates and corresponding confidence intervals (the regression coefficients in the truncated negative binomial components and the true stayer proportion estimates in each joint area together with their corresponding confidence intervals) were similar to those displayed in Table 1. Comparison of likelihood values suggested a preference towards a gamma distributional assumption for *U*_*i*_, hence demonstrating its usefulness in this context.

For comparative purposes, trivariate models with truncated negative binomial ( $\pi_{1}^{k}:=0\forall k$, TNB model) and mover‐stayer truncated Poisson ( $\theta^{k}=\theta_{0}^{k}:=0\forall k$, TM‐SP model) margins were also fitted. Tables 2 and 3 displays the results from the TNB and TM‐SP models, respectively. Rather reassuringly, the TNB model demonstrates similar results to the full model regarding the direction and significance of associations between covariates and outcomes. However, the effect sizes corresponding to the significant associations from the TNB model are generally inflated in comparison with the full model. The estimated values of *θ* ^*k*^ and $\theta^{k_{0}}$ for each *k* are also seen to be larger in the TNB model, which is unsurprising because these parameters now also reflect the variability from the existence of stayers. In contrast, the results from the TM‐SP model are less similar to the full model. Specifically, the number of active large joints are now seen to be strongly and positively associated with damage progression in all three joint areas and the number of damaged large joints is strongly and negatively associated with damaged large joints progression. The strong association between the number of damaged hand joints and damage progression in all three joint areas has also been greatly attenuated, whilst the stayer proportion estimates are greatly inflated. It is also interesting to note that $\hat{\gamma }$ takes a smaller value in the TM‐SP model (in comparison with the full model), which suggest that there is less correlation between the distribution of stayers in the different joint areas when heterogeneity is not taken into account in the damage progression models, after adjusting for covariates. The log‐likelihood values strongly indicate, even after accounting for the 4 less parameters from the TNB model and the 6 less parameters from the TM‐SP model, that the full model is to be preferred.

**Table 2:** Parameter estimates related to associations with damaged joint counts and stayer probability estimates obtained from fitting the TNB model to 1194 psoriatic arthritis patients.

Damage Progression	Hand Joints	Foot Joints	Large Joints
Attained number of
damaged hand joints	0.19 (0.15, 0.22)	0.17 (0.098, 0.24)	0.038 (0.018, 0.059)
Current number of
active hand joints	0.11 (0.08, 0.14)	0.015 (−0.023, 0.052)	0.004 (−0.026, 0.034)
Attained number of
damaged foot joints	0.0084 (−0.013, 0.029)	0.14 (0.085, 0.19)	0.022 (−0.0013, 0.045)
Current number of
active foot joints	−0.003 (−0.033, 0.027)	0.12 (0.069, 0.16)	−0.056 (−0.093, −0.019)
Attained number of
damaged large joints	0.026 (−0.043, 0.095)	0.15 (0.033, 0.28)	0.065 (−0.0016, 0.13)
Current number of
active large joints	0.018 (−0.045, 0.081)	0.043 (−0.041, 0.13)	0.32 (0.25, 0.4)
*λ*_0_	0.076 (0.065, 0.088)	0.11 (0.092, 0.13)	0.037 (0.031, 0.044)
$\lambda_{0}^{0}$	0.41 (0.31, 0.54)	0.37 (0.27, 0.51)	0.065 (0.052, 0.081)
*θ*	11.19 (10.01, 12.52)	17.55 (15.89, 19.4)	10.27 (8.4, 12.53)
*θ* ^0^	8.27 (7.04, 9.72)	10.1 (8.51, 12)	5.88 (4.52, 7.65)
Log‐likelihood	−13738.69		

**Table 3:** Parameter estimates related to associations with damaged joint counts and stayer probability estimates obtained from fitting the TM‐SP model to 1194 psoriatic arthritis patients. The P(Stayer) estimates were calculated as $\pi_{1}^{k*}({\hat{\alpha }}_{1},\hat{\gamma })$ for *k* = *h*, *f* and *l*. Standard errors for these quantities were calculated using the delta method.

Damage progression	Hand joints	Foot joints	Large joints
Attained number of
damaged hand joints	0.048 (0.04, 0.057)	0.049 (0.042, 0.056)	0.027 (0.013, 0.04)
Current number of
active hand joints	0.073 (0.063, 0.082)	−0.0031 (−0.013, 0.0063)	−0.0049 (−0.024, 0.014)
Attained number of
damaged foot joints	0.0029 (−0.0056, 0.011)	−0.041 (−0.051, −0.031)	0.012 (−0.0032, 0.028)
Current number of
active foot joints	−0.0051 (−0.017, 0.0072)	0.055 (0.046, 0.064)	−0.04 (−0.065, −0.015)
Attained number of
damaged large joints	−0.0027 (−0.028, 0.022)	0.059 (0.035, 0.083)	−0.077 (−0.12, −0.033)
Current number of
active large joints	0.032 (0.0062, 0.057)	0.068 (0.047, 0.09)	0.21 (0.18, 0.25)
*λ*_0_	0.21 (0.19, 0.22)	0.35 (0.33, 0.37)	0.094 (0.082, 0.11)
$\lambda_{0}^{0}$	0.36 (0.34, 0.38)	0.32 (0.31, 0.34)	0.099 (0.089, 0.11)
P(Stayer)	0.52 (0.49, 0.55)	0.51 (0.47, 0.54)	0.51 (0.47, 0.55)
*γ*	1.26 (1.04, 1.53)		
Log‐likelihood	−19550.19		

## 5 Simulation study

This section evaluates the empirical performance of the full model through simulation studies. The simulation studies consist of simulating data from the full model and then refitting the same model structure to the simulated data. This will determine if the parameters of interest can be reasonably estimated. Specifically if the regression coefficients corresponding to the dynamic covariates, that is the relationship between the damaged joints counting processes, and the stayer proportions can be well estimated. In the simulation studies, 400 data sets were generated from the full model with each data set containing patients with 18 clinic visits and 6 months inter‐visit time, thus reflecting the average patient in the PsA data. For simplicity, all patients are assumed to have entered the clinic with no damaged joints (in any joint area), and therefore, the models for $D_{i0*}^{k}$ are not applicable. Furthermore, the number of currently active joints are not considered. Simulations were performed with the number of patients in each data set being fixed at 200, 500 and 800.

Simulation from the full model was performed as follows. Firstly, a value $u_{i}^{s}$ was simulated from a gamma distribution with unit mean and variance *γ* for each patient. Then for each joint area, a Bernoulli variable $C_{i}^{\mathrm{ks}}$ was simulated with success probability $\exp\left(-\overset{s}{\underset{i}{u}}\exp(\alpha^{k})\right)$ to determine if that simulated patient was a stayer ( $C_{i}^{\mathrm{ks}}=0$) or a mover ( $C_{i}^{\mathrm{ks}}=1$) in joint area *k*. Simulated stayers in joint area *k* were such that their simulated outcomes $n_{\mathrm{ij}}^{\mathrm{ks}}=0$ for all time intervals, whilst for movers $n_{\mathrm{ij}+1}^{\mathrm{ks}}=d_{\mathrm{ij}}^{\mathrm{ks}}+n_{\mathrm{ij}}^{\mathrm{ks}}$ and $n_{i0}^{\mathrm{ks}}=0$. The damage joints increment $d_{\mathrm{ij}}^{\mathrm{ks}}$ were obtained by simulating from a negative binomial distribution with mean $0.5\lambda_{0}^{k}\exp\left(\overset{k}{\underset{h}{\beta }}\overset{\mathrm{hs}}{\underset{\mathrm{ij}}{n}}+\beta_{f}^{k}n_{\mathrm{ij}}^{\mathrm{fs}}+\beta_{l}^{k}n_{\mathrm{ij}}^{\mathrm{ls}}\right)$ and dispersion parameter $\theta_{0}^{k}$, which had been truncated so that $0\leq d_{\mathrm{ij}}^{\mathrm{ks}}\leq T^{k}-n_{\mathrm{ij}}^{\mathrm{ks}}$. Simulating from such a distribution was performed by simulating from a multinomial distribution with categories $\left\{0,\ldots ,T^{k}-n_{\mathrm{ij}}^{\mathrm{ks}}\right\}$, where the category probabilities were calculated from the specified truncated negative binomial distribution.

Table 4 displays the true values and results of the simulation studies for the parameters of interest. The other true parameter values were set as $\lambda_{0}^{h}=0.37$, $\lambda_{0}^{f}=0.61$, $\lambda_{0}^{l}=0.14$, *θ* ^*h*^=8, *θ* ^*f*^=13, *θ* ^*l*^=8 and *γ* = 3.9. Parameter values were chosen to reflect similar characteristics to those observed in Table 1 (results from fitting the full model to our PsA data). When there are 500 or 800 patients in each data set, Table 4 demonstrates that the model fitting procedure produces little empirical bias because mean estimated parameters are similar to their true values. More empirical bias is observed when only 200 patients are in each data set, although the bias is generally not substantial. The mean estimated standard error and the standard deviation for each estimated parameter is seen to be similar in all three scenarios, which indicate the reasonableness of asymptotic approximations even with relatively small sample sizes.

**Table 4:** Simulation results displaying mean parameter estimates (mean estimated standard error, standard deviation) for the scenarios where the number of patients in each data set are 200, 500 and 800, respectively. Only the parameters of interest are reported, although $\lambda_{0}^{k}$, *θ* ^*k*^ and *γ* were also estimated for each *k*.

	True	200 Patients	500 Patients	800 Patients
Hand joints
$\beta_{h}^{h}$	0.1	0.094 (0.037, 0.04)	0.098 (0.023, 0.023)	0.099 (0.018, 0.018)
$\beta_{f}^{h}$	0	−0.00064 (0.022, 0.022)	0.00025 (0.013, 0.014)	−0.00073 (0.011, 0.01)
$\beta_{l}^{h}$	0	−0.0079 (0.089, 0.09)	−0.0019 (0.055, 0.056)	−0.0029 (0.043, 0.042)
$\pi_{1}^{h}$	0.37	0.37 (0.045, 0.044)	0.37 (0.028, 0.027)	0.37 (0.022, 0.023)
Foot joints
$\beta_{h}^{f}$	0.08	0.087 (0.056, 0.06)	0.081 (0.034, 0.036)	0.08 (0.027, 0.027)
$\beta_{f}^{f}$	0.04	0.044 (0.043, 0.046)	0.042 (0.026, 0.028)	0.04 (0.021, 0.019)
$\beta_{l}^{f}$	0.1	0.11 (0.13, 0.13)	0.1 (0.075, 0.074)	0.098 (0.059, 0.058)
$\pi_{1}^{f}$	0.31	0.3 (0.041, 0.042)	0.3 (0.026, 0.024)	0.3 (0.02, 0.021)
Large joints
$\beta_{h}^{l}$	0.03	0.023 (0.042, 0.046)	0.029 (0.025, 0.025)	0.027 (0.02, 0.02)
$\beta_{f}^{l}$	0	−0.0028 (0.027, 0.028)	−0.00042 (0.017, 0.017)	0.00092 (0.013, 0.013)
$\beta_{l}^{l}$	0	−0.03 (0.11, 0.18)	−0.011 (0.069, 0.071)	−0.0056 (0.054, 0.057)
$\pi_{1}^{l}$	0.29	0.29 (0.062, 0.069)	0.29 (0.04, 0.04)	0.29 (0.031, 0.031)

## 6 Model for trivariate mover‐clinic‐induced stayer‐true stayer damaged joints counting processes

In the previous sections, the stayer property was thought of as inherent. It is plausible to think that this property could also have arisen from entry into the clinic; possibly through treatment/management strategies employed by the clinicians. Such patients would therefore be susceptible to damage before clinic entry and ‘immune’ thereafter (clinic‐induced stayers). Hence, information before clinic entry would not contribute towards estimating these patients' clinic‐induced stayer probabilities. To distinguish between these subgroups, true stayers (patients who are inherent stayers) and clinic‐induced stayers are considered separately.

### 6.1 Model

Let $C_{i1}^{k}$ be again the partially observable binary variable such that $C_{i1}^{k}=0$ if patient *i* is a true stayer in joint area *k* and $C_{i1}^{k}=1$ otherwise. If this patient in this joint area is a mover before clinic entry, that is $C_{i1}^{k}=1$, let $C_{i2}^{k}$ be another partially observable binary variable such that $C_{i2}^{k}=0$ if this patient becomes a clinic‐induced stayer in joint area *k* and $C_{i2}^{k}=1$ otherwise. These variables are again partially observable for reasons previously discussed. The probability of these events can then be estimated as

$$\pi_{i1}^{k}:=\pi_{i1}^{k}\left(\alpha_{1}^{k}|z_{i⋆}^{k},u_{i}\right)=P\;\left(C_{i1}^{k}=0|z_{i⋆}^{k},u_{i}\right)=1-\exp\;\left(-u_{i}\exp\;\left(\overset{k^{\prime }}{\underset{1}{\alpha }}z_{i⋆}^{k}\right)\right),$$

and

$$\pi_{i2}^{k}:=\pi_{i2}^{k}\left(\alpha_{2}^{k}|z_{i*}^{k},v_{i}\right)=P\;\left(C_{i2}^{k}=0|C_{i1}^{k}=1;z_{i*}^{k},v_{i}\right)=1-\exp\;\left(-v_{i}\exp\;\left(\overset{k^{\prime }}{\underset{2}{\alpha }}z_{i*}^{k}\right)\right),$$

where again $z_{i⋆}^{k}$, $z_{i*}^{k}$ and $\alpha_{1}^{k}$, $\alpha_{2}^{k}$ are column vectors of covariates and regression coefficients, respectively, and *u*_*i*_, *v*_*i*_ are realisations of patient‐level random effects *U*_*i*_, *V*_*i*_, which are assumed independent. Note that *V*_*i*_ is only relevant in joint area *k* if patient *i* is not a true stayer in this joint area. These random effects again represent the characteristic that the stayer probabilities in the different joint areas, both true and clinic‐induced, are likely to be more similar within patients. The likelihood contribution $L_{i}^{k}(\Theta |u_{i},v_{i})$ from patient *i* in joint area *k*, is firstly, if $n_{im_{i}}^{k}=0$;

$$\begin{array}{c} \;P\;\left(N_{i0}^{k}=0,\ldots ,N_{im_{i}}^{k}=0|N_{i0^{*}}^{k}=0;u_{i},v_{i}\right)\\ =P\;\left(D_{i0^{*}}^{k}=0,\ldots ,D_{im_{i}-1}^{k}=0|C_{i1}^{k}=0\right)P\;\left(C_{i1}^{k}=0|u_{i}\right)\\ \;+P\;\left(D_{i0^{*}}^{k}=0,\ldots ,D_{im_{i}-1}^{k}=0|C_{i1}^{k}=1\right)P\;\left(C_{i1}^{k}=1|u_{i}\right)\\ =P\;\left(D_{i0^{*}}^{k}=0,\ldots ,D_{im_{i}-1}^{k}=0|C_{i1}^{k}=0\right)P\;\left(C_{i1}^{k}=0|u_{i}\right)\\ \;+P\;\left(D_{i0^{*}}^{k}=0,\ldots ,D_{im_{i}-1}^{k}=0|C_{i2}^{k}=0,C_{i1}^{k}=1\right)P\;\left(C_{i2}^{k}=0|C_{i1}^{k}=1;v_{i}\right)\left(1-\pi_{i1}^{k}\right)\\ \;+P\;\left(D_{i0^{*}}^{k}=0,\ldots ,D_{im_{i}-1}^{k}=0|C_{i2}^{k}=1,C_{i1}^{k}=1\right)P\;\left(C_{i2}^{k}=1|C_{i1}^{k}=1;v_{i}\right)\left(1-\pi_{i1}^{k}\right)\\ =\pi_{i1}^{k}+\left(1-\pi_{i1}^{k}\right)P\;\left(D_{i0^{*}}^{k}=0|N_{i0^{*}}^{k}=0\right)\left\{\pi_{i2}^{k}+\left(1-\pi_{i2}^{k}\right)\overset{m_{i}-1}{\underset{j=0}{\prod }}P\;\left(D_{\mathrm{ij}}^{k}=0|{\left\{N_{\mathrm{ij}}^{k}=n_{\mathrm{ij}}^{k}\right\}}_{k=h,f,l}\right)\right\}. \end{array}$$

Secondly, if $n_{im_{i}}^{k}\neq 0$ and $n_{im_{i}}^{k}=n_{i0}^{k}$;

$$\begin{array}{c} \;P\;\left(N_{i0}^{k}=n_{i0}^{k},\ldots ,N_{im_{i}}^{k}=n_{i0}^{k}|N_{i0^{*}}^{k}=0;u_{i},v_{i}\right)\\ =P\;\left(D_{i0^{*}}^{k}=n_{i0}^{k},D_{i0}^{k}=0,\ldots ,D_{im_{i}-1}^{k}=0|C_{i1}^{k}=0\right)P\;\left(C_{i1}^{k}=0|u_{i}\right)\\ \;+P\;\left(D_{i0^{*}}^{k}=n_{i0}^{k},D_{i0}^{k}=0,\ldots ,D_{im_{i}-1}^{k}=0|C_{i1}^{k}=1\right)P\;\left(C_{i1}^{k}=1|u_{i}\right)\\ =P\;\left(D_{i0^{*}}^{k}=n_{i0}^{k},D_{i0}^{k}=0,\ldots ,D_{im_{i}-1}^{k}=0|C_{i2}^{k}=0,C_{i1}^{k}=1\right)P\;\left(C_{i2}^{k}=0|C_{i1}^{k}=1;v_{i}\right)\left(1-\pi_{i1}^{k}\right)\\ \;+P\;\left(D_{i0^{*}}^{k}=n_{i0}^{k},D_{i0}^{k}=0,\ldots ,D_{im_{i}-1}^{k}=0|C_{i2}^{k}=1,C_{i1}^{k}=1\right)P\;\left(C_{i2}^{k}=1|C_{i1}^{k}=1;v_{i}\right)\left(1-\pi_{i1}^{k}\right)\\ =\left(1-\pi_{i1}^{k}\right)P\;\left(D_{i0^{*}}^{k}=n_{i0}^{k}|N_{i0^{*}}^{k}=0\right)\left\{\pi_{i2}^{k}+\left(1-\pi_{i2}^{k}\right)\overset{m_{i}-1}{\underset{j=0}{\prod }}P\;\left(D_{\mathrm{ij}}^{k}=0|{\left\{N_{\mathrm{ij}}^{k}=n_{\mathrm{ij}}^{k}\right\}}_{k=h,f,l}\right)\right\}, \end{array}$$

and finally, if $n_{im_{i}}^{k}>n_{i0}^{k}$;

$$\begin{array}{c} \;P\;\left(N_{i0}^{k}=n_{i0}^{k},\ldots ,N_{im_{i}}^{k}=n_{im_{i}}^{k}|N_{i0^{*}}^{k}=0;u_{i},v_{i}\right)\\ =P\;\left(D_{i0^{*}}^{k}=n_{i0}^{k},D_{i0}^{k}=d_{i0}^{k},\ldots ,D_{im_{i}-1}^{k}=d_{im_{i-1}}^{k}|C_{i1}^{k}=0\right)P\;\left(C_{i1}^{k}=0|u_{i}\right)\\ \;+P\;\left(D_{i0^{*}}^{k}=n_{i0}^{k},D_{i0}^{k}=d_{i0}^{k},\ldots ,D_{im_{i}-1}^{k}=d_{im_{i-1}}^{k}|C_{i1}^{k}=1\right)P\;\left(C_{i1}^{k}=1|u_{i}\right)\\ =P\;\left(D_{i0^{*}}^{k}=n_{i0}^{k},D_{i0}^{k}=d_{i0}^{k},\ldots ,D_{im_{i}-1}^{k}=d_{im_{i-1}}^{k}|C_{i2}^{k}=0,C_{i1}^{k}=1\right)P\;\left(C_{i2}^{k}=0|C_{i1}^{k}=1;v_{i}\right)\left(1-\pi_{i1}^{k}\right)\\ \;+P\;\left(D_{i0^{*}}^{k}=n_{i0}^{k},D_{i0}^{k}=d_{i0}^{k},\ldots ,D_{im_{i}-1}^{k}=d_{im_{i-1}}^{k}|C_{i2}^{k}=1,C_{i1}^{k}=1\right)P\;\left(C_{i2}^{k}=1|C_{i1}^{k}=1;v_{i}\right)\left(1-\pi_{i1}^{k}\right)\\ =\left(1-\pi_{i1}^{k}\right)\left(1-\pi_{i2}^{k}\right)P\;\left(D_{i0^{*}}^{k}=n_{i0}^{k}|N_{i0^{*}}^{k}=0\right)\overset{m_{i}-1}{\underset{j=0}{\prod }}P\;\left(D_{\mathrm{ij}}^{k}=0|{\left\{N_{\mathrm{ij}}^{k}=n_{\mathrm{ij}}^{k}\right\}}_{k=h,f,l}\right). \end{array}$$

By then assuming independence between the damaged joints counting processes given the patient‐level random effects and dynamic covariates, the likelihood contribution from patient *i* is

$$L_{i}(\Theta )=\overset{\infty }{\underset{0}{\int }}\overset{\infty }{\underset{0}{\int }}L_{i}^{h}(\Theta |u_{i},v_{i})L_{i}^{f}(\Theta |u_{i},v_{i})L_{i}^{l}(\Theta |u_{i},v_{i})g(u_{i}|\gamma_{1})g(v_{i}|\gamma_{2})\;du_{i}\;dv_{i},$$

where *g*(*u*_*i*_|*γ*_1_) and *g*(*v*_*i*_|*γ*_2_) are random effect densities with unit means and variances *γ*_1_ and *γ*_2_, respectively. A closed form solution to the integrations in *L*_*i*_(Θ) can again be achieved by using the technique of Section 3.2; see Appendix A for more details. Note that the model of Section 3 is retained if $\pi_{i2}^{k}=0$ for all *i* and *k*.

### 6.2 Exploring the existence of a clinic‐induced and true stayer subpopulation

Intercept only models for $\pi_{i1}^{k}$ and $\pi_{i2}^{k}$ were fitted in order to investigate the existence of a true and clinic‐induced stayer subpopulation in each joint area. Initially, the random effects *U*_*i*_ and *V*_*i*_ were assumed gamma distributed. The resulting marginal estimates $\pi_{2}^{k*}\left({\hat{\alpha }}_{2}^{k},{\hat{\gamma }}_{2}\right)$ (after integrating out *v*_*i*_ but still conditional on $C_{i1}^{k}=1$) were small for each *k*. There was also a lot of uncertainty about the estimate ${\hat{\gamma }}_{2}$, which therefore transfered to $\pi_{2}^{k*}\left({\hat{\alpha }}_{2}^{k},{\hat{\gamma }}_{2}\right)$. When *γ*_2_>1, *g*(*v*_*i*_|*γ*_2_)→*∞* as *v*_*i*_→0, hence, small values of $\pi_{2}^{k}$ are likely to result from $\alpha_{2}^{k}\approx -\infty$ or *v*_*i*_≈0. It is therefore unsurprising that identifiability issues arose. An alternative for *V*_*i*_ is the inverse Gaussian distribution with unit mean and variance *ψ*. In this case, *g*(*v*_*i*_|*ψ*)→0 as *v*_*i*_→0, hence, small values of $\pi_{2}^{k}$ are less likely to result from *v*_*i*_≈0. The model was then fitted for this distributional assumption of *V*_*i*_, and this produced a similar likelihood value to the case when *V*_*i*_ was assumed gamma distributed. Note that marginally

$$\pi_{2}^{k*}:=\pi_{2}^{k*}\left(\alpha_{2}^{k},\psi \right)=1-\exp\;\left(\frac{1}{\psi }\left(1-\sqrt{1+2\psi \exp\;\left(\overset{k}{\underset{2}{\alpha }}\right)}\right)\right).$$

**Figure 3:**
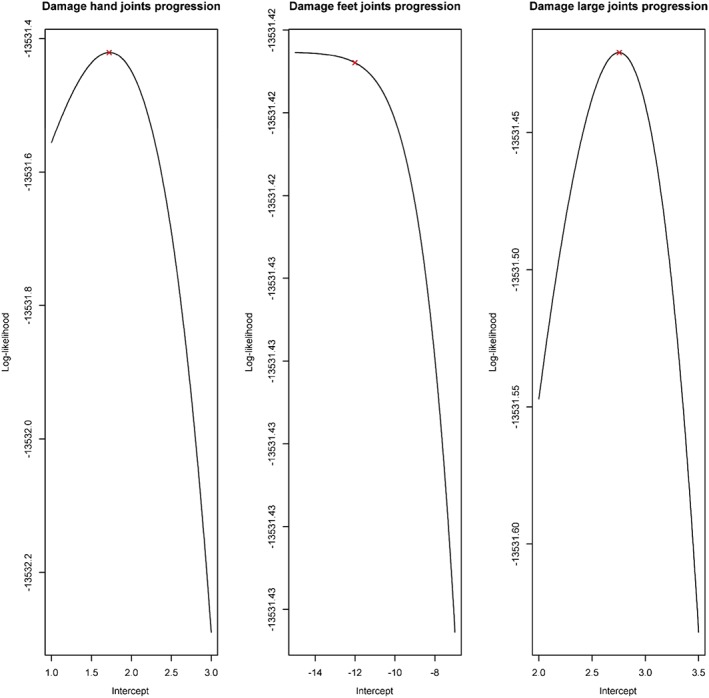
Plots of the profile log‐likelihood for $\alpha_{2}^{k}$, *k* = *h*, *f* and *l*. The cross indicates the point at which the numerical optimisation procedure converged.

**Figure 4:**
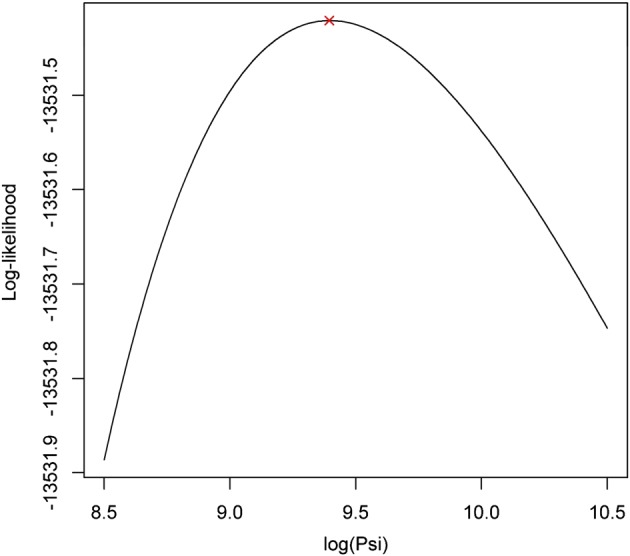
Plot of the profile log‐likelihood for log(*ψ*). The cross indicates the point at which the numerical optimisation procedure converged.

Figures 3 and 4 display the profile log‐likelihood for $\alpha_{2}^{h}$, $\alpha_{2}^{f}$, $\alpha_{2}^{l}$ and log(*ψ*). These plots suggest that the optimisation procedure was able to identify the parameters $\alpha_{2}^{h}$, $\alpha_{2}^{l}$ and log(*ψ*) as it converged at the maximum of their respective profile log‐likelihoods. From Figure 3, it is also clear that the maximum of the profile log‐likelihood for $\alpha_{2}^{f}$ occurs at a large negative value. It is therefore likely that a clinic‐induced subpopulation does not exist in the foot joints. The maximum likelihood estimates for $\pi_{2}^{h*}$ and $\pi_{2}^{l*}$ were 0.03 (SE = 0.0228) and 0.0498 (SE = 0.0508), respectively. The standard errors were approximated using the delta method. Because there is large uncertainty associated with these parameter estimates, there is no convincing evidence to suggest clinic‐induced stayer subpopulations exist in the hand and large joints. In contrast and similarly to the previous results, the estimates and corresponding confidence intervals for $\pi_{1}^{k*}$ were 0.36 (0.313, 0.407), 0.308 (0.264, 0.352) and 0.267 (0.193, 0.341) for *k* = *h*, *f* and *l*, respectively. As all of the confidence intervals are again far from zero, there is reasonable evidence to suggest that a subpopulation of true stayers exist in all three joint areas.

## 7 Discussion

This research was clinically motivated by the need to understand the relationship between damage progression in the hands, feet and large joints, under the assumption that the stayer property is inherent, and then relaxing this assumption by allowing for the possibility of a clinic‐induced stayer population. For these purposes, this paper proposed novel trivariate (two‐level) mover‐stayer models consisting of mover‐stayer/mover‐clinic‐induced stayer‐true stayer truncated negative binomial margins, and patient‐level dynamic covariates and random effects to account for within‐patient correlation. These models are appealing because the measures of associations between the counting processes are captured using regression coefficients, and the marginal likelihood can be computed analytically even when random effects are used to correlate the stayer components. The former allows asymmetric associations to be accommodated, whilst the latter facilitates the model fitting procedure. When applied to the PsA data, asymmetric associations between the three damaged joints counting processes resulted. A particular interesting observation was the significant positive associations between the attained number of damaged hand joints and damage progression in all three damaged joints areas. Confirmatory evidence of the local effect of active joints was also seen. Furthermore, little evidence was found for the existence of clinic‐induced stayer populations in any of the three joint areas.

In the proposed methodology, a clinic‐induced stayer was investigated by assuming ‘immunity’ to damage occurred at the point of clinic entry. Whilst this may be a reasonable approximation for patients who have a controllable amount of disease activity, it is likely to be inaccurate for patients who have severe disease activity at clinic entry. A fairer assessment of a clinic‐induced stayer population might therefore allow such patients to gain ‘immunity’ to damage at other time points whilst in the clinic. This can most conveniently be achieved by modelling, for each patient, the rate of becoming a clinic‐induced stayer, as opposed to modelling the explicit proportion of such a population. Such an analysis would however have to account for the uncertainty of not knowing if, in addition to when, a patient has become a clinic‐induced stayer, and therefore, is likely to require the development of substantially new statistical methodology.

## Appendix A

### A.1

We demonstrate how the marginal likelihood of the trivariate mover‐clinic‐induced stayer‐true stayer counting process model can be computed analytically. Let $c_{i1*}^{k}=0$ if $n_{im_{i}}^{k}=0$ and $c_{i1*}^{k}=1$ otherwise. If $c_{i1*}^{k}=1$, let $c_{i2*}^{k}=0$ if $n_{im_{i}}^{k}=n_{i0}^{k}$ and $c_{i2*}^{k}=1$ otherwise. The likelihood contribution from patient *i* in joint area *k* can again be rearranged as

$$\begin{array}{cc} L_{i}^{k}(\Theta |u_{i},v_{i}) & ={\left\{1-\exp\;\left(-u_{i}\omega_{i1}^{k}\right)\left\{1-\overset{k}{\underset{i1}{P}}+P_{i1}^{k}\left(1-P_{i2}^{k}\right)\exp\;\left(-v_{i}\omega_{i2}^{k}\right)\right\}\right\}}^{1-c_{i1*}^{k}}\\ \;\times {\left\{\exp\;\left(-u_{i}\omega_{i1}^{k}\right)\overset{k}{\underset{i1}{P}}{\left\{1-\exp\;\left(-v_{i}\omega_{i2}^{k}\right)\left(1-\overset{k}{\underset{i2}{P}}\right)\right\}}^{1-c_{i2*}^{k}}{\left\{\exp\;\left(-v_{i}\omega_{i2}^{k}\right)\overset{k}{\underset{i2}{P}}\right\}}^{c_{i2*}^{k}}\right\}}^{c_{i1*}^{k}}, \end{array}$$

where

$$\begin{array}{cc} P_{i1}^{k} & =P\;\left(D_{i0^{*}}^{k}=n_{i0}^{k}|N_{i0^{*}}^{k}=0\right)\\ P_{i2}^{k} & =\overset{m_{i-1}}{\underset{j=0}{\prod }}P\;\left(D_{\mathrm{ij}}^{k}=d_{\mathrm{ij}}^{k}|{\left\{N_{\mathrm{ij}}^{k}=n_{\mathrm{ij}}^{k}\right\}}_{k=h,f,l}\right)\\ \omega_{i1}^{k} & =\exp\;\left(\overset{k^{\prime }}{\underset{j}{\alpha }}z_{i⋆}^{k}\right)\\ \omega_{i2}^{k} & =\exp\;\left(\overset{k^{\prime }}{\underset{j}{\alpha }}z_{i*}^{k}\right). \end{array}$$

When $c_{i1*}^{k}=1$ for each *k*,

$$\begin{array}{cc} L_{i}(\Theta |u_{i},v_{i}) & =\underset{k=h,f,l}{\prod }\exp\;\left(-u_{i}\omega_{i1}^{k}\right)\overset{k}{\underset{i1}{P}}{\left\{1-\exp\;\left(-v_{i}\omega_{i2}^{k}\right)\left(1-\overset{k}{\underset{i2}{P}}\right)\right\}}^{1-c_{i2*}^{k}}{\left\{\exp\;\left(-v_{i}\omega_{i2}^{k}\right)\overset{k}{\underset{i2}{P}}\right\}}^{c_{i2*}^{k}}\\ =\left[\;\underset{k=h,f,l}{\prod }P_{i1}^{k}\;\right]\;\exp\;\left(\;\;-u_{i}\underset{k=h,f,l}{\sum }\omega_{i1}^{k}\;\;\right)\underset{k=h,f,l}{\prod }\;{\left\{\;1\;-\exp\;\left(-v_{i}\overset{k}{\underset{i2}{\omega }}\right)\;\left(\;1-\overset{k}{\underset{i2}{P}}\right)\;\right\}}^{1-\overset{k}{\underset{i2*}{c}}}\;{\left\{\exp\;\left(-v_{i}\overset{k}{\underset{i2}{\omega }}\right)\overset{k}{\underset{i2}{P}}\right\}}^{\overset{k}{\underset{i2*}{c}}}\\ =\left[\underset{k=h,f,l}{\prod }P_{i1}^{k}\right]\overset{1-c_{i2*}^{h}}{\underset{r_{h}=0}{\sum }}\overset{1-c_{i2*}^{f}}{\underset{r_{f}=0}{\sum }}\overset{1-c_{i2*}^{l}}{\underset{r_{l}=0}{\sum }}{\left(-1\right)}^{r_{h}+r_{f}+r_{l}}\left[\underset{k=h,f,l}{\prod }{\left\{P_{i2}^{k}\right\}}^{c_{i2*}^{k}}{\left\{1-P_{i2}^{k}\right\}}^{r_{k}\left(1-c_{i2*}^{k}\right)}\right]\\ \;\times \exp\;\left(-u_{i}\underset{k=h,f,l}{\sum }\omega_{i1}^{k}-v_{i}\underset{k=h,f,l}{\sum }\omega_{i2}^{k}\left(c_{i2*}^{k}+r_{k}\left(1-c_{i2*}^{k}\right)\right)\right). \end{array}$$

Without loss of generality, let $c_{i1*}^{h}=0$ and $c_{i1*}^{f}=c_{i1*}^{l}=1$, then

$$\begin{array}{cc} L_{i}(\Theta |u_{i},v_{i}) & =\left\{1-\exp\;\left(-u_{i}\omega_{i1}^{h}\right)\left\{1-\overset{h}{\underset{i1}{P}}+P_{i1}^{h}\left(1-P_{i2}^{h}\right)\exp\;\left(-v_{i}\omega_{i2}^{h}\right)\right\}\right\}\\ \;\times \left[\underset{k=f,l}{\prod }P_{i1}^{k}\right]\overset{1-c_{i2*}^{f}}{\underset{r_{f}=0}{\sum }}\overset{1-c_{i2*}^{l}}{\underset{r_{l}=0}{\sum }}{\left(-1\right)}^{r_{f}+r_{l}}\left[\underset{k=f,l}{\prod }{\left\{P_{i2}^{k}\right\}}^{c_{i2*}^{k}}{\left\{1-P_{i2}^{k}\right\}}^{r_{k}\left(1-c_{i2*}^{k}\right)}\right]\\ \;\times \exp\;\left(-u_{i}\underset{k=f,l}{\sum }\omega_{i1}^{k}-v_{i}\underset{k=f,l}{\sum }\omega_{i2}^{k}\left(c_{i2*}^{k}+r_{k}\left(1-c_{i2*}^{k}\right)\right)\right)\\ =\left[\underset{k=f,l}{\prod }P_{i1}^{k}\right]\overset{1-c_{i2*}^{f}}{\underset{r_{f}=0}{\sum }}\overset{1-c_{i2*}^{l}}{\underset{r_{l}=0}{\sum }}{\left(-1\right)}^{r_{f}+r_{l}}\left[\underset{k=f,l}{\prod }{\left\{P_{i2}^{k}\right\}}^{c_{i2*}^{k}}{\left\{1-P_{i2}^{k}\right\}}^{r_{k}\left(1-c_{i2*}^{k}\right)}\right]\\ \;\times \exp\;\left(-v_{i}\underset{k=f,l}{\sum }\omega_{i2}^{k}\left(c_{i2*}^{k}+r_{k}\left(1-c_{i2*}^{k}\right)\right)\right)\left\{\exp\;\left(-u_{i}\underset{k=f,l}{\sum }\overset{k}{\underset{i1}{\omega }}\right)\right.\\ \left.\;-(1-P_{i1}^{h})\exp\;\left(-u_{i}\underset{k=h,f,l}{\sum }\omega_{i1}^{k}\right)-\overset{h}{\underset{i1}{P}}\left(1-P_{i2}^{h}\right)\exp\;\left(-u_{i}\underset{k=h,f,l}{\sum }\omega_{i1}^{k}-v_{i}\omega_{i2}^{h}\right)\right\}. \end{array}$$

Similarly, without loss of generality, let $c_{i1*}^{h}=c_{i1*}^{f}=0$ and $c_{i1*}^{l}=1$, then

$$\begin{array}{c} L_{i}(\Theta |u_{i},v_{i})=\underset{k=h,f}{\prod }\left\{1-\exp\;\left(-u_{i}\omega_{i1}^{k}\right)\left\{1-\overset{k}{\underset{i1}{P}}+P_{i1}^{k}\left(1-P_{i2}^{k}\right)\exp\;\left(-v_{i}\omega_{i2}^{k}\right)\right\}\right\}\\ \;\times \exp\;\left(-u_{i}\omega_{i1}^{l}\right)\overset{l}{\underset{i1}{P}}{\left\{1-\exp\;\left(-v_{i}\omega_{i2}^{l}\right)\left(1-\overset{l}{\underset{i2}{P}}\right)\right\}}^{1-c_{i2*}^{l}}{\left\{\exp\;\left(-v_{i}\omega_{i2}^{l}\right)\overset{l}{\underset{i2}{P}}\right\}}^{c_{i2*}^{l}}\\ =\overset{1}{\underset{r_{h}=0}{\sum }}\overset{1}{\underset{r_{f}=0}{\sum }}{\left(-1\right)}^{r_{h}+r_{f}}\exp\;\left(-u_{i}\underset{k=h,f}{\sum }r_{k}\omega_{i1}^{k}\right)\underset{k=h,f}{\prod }{\left\{1-\overset{k}{\underset{i1}{P}}+\overset{k}{\underset{i1}{P}}\left(1-\overset{k}{\underset{i2}{P}}\right)\exp\;\left(-v_{i}\overset{k}{\underset{i2}{\omega }}\right)\right\}}^{r_{k}}\\ \;\times \exp\;\left(-u_{i}\omega_{i1}^{l}\right)\overset{l}{\underset{i1}{P}}\overset{1-c_{i2*}^{l}}{\underset{r_{l}=0}{\sum }}{\left(-1\right)}^{r_{l}}{\left\{P_{i2}^{l}\right\}}^{c_{i2*}^{l}}{\left\{1-P_{i2}^{l}\right\}}^{r_{l}\left(1-c_{i2*}^{l}\right)}\exp\;\left(-v_{i}\omega_{i2}^{l}\left(c_{i2*}^{l}+r_{l}\left(1-c_{i2*}^{l}\right)\right)\right)\\ =\overset{1}{\underset{r_{h}=0}{\sum }}\overset{1}{\underset{r_{f}=0}{\sum }}\overset{1-c_{i2*}^{l}}{\underset{r_{l}=0}{\sum }}{\left(-1\right)}^{r_{h}+r_{f}+r_{l}}\exp\;\left(-u_{i}\left(\underset{k=h,f}{\sum }r_{k}\omega_{i1}^{k}+\omega_{i1}^{l}\right)\right)\overset{l}{\underset{i1}{P}}{\left\{P_{i2}^{l}\right\}}^{c_{i2*}^{l}}{\left\{1-P_{i2}^{l}\right\}}^{r_{l}\left(1-c_{i2*}^{l}\right)}\\ \;\times \overset{r_{h}}{\underset{r_{h}^{\prime }=0}{\sum }}\overset{r_{f}}{\underset{r_{f}^{\prime }=0}{\sum }}\underset{k=h,f}{\prod }{\left\{\;{\left[1-P_{i1}^{k}\right]}^{1-r_{k}^{\prime }}{\left[P_{i1}^{k}\left(1-P_{i2}^{k}\right)\right]}^{r_{k}^{\prime }}\right\}}^{r_{k}}\exp\;\left(\;\;-v_{i}\left(\underset{k=h,f}{\sum }r_{k}^{\prime }\omega_{i2}^{k}+\omega_{i2}^{l}\left(c_{i2*}^{l}+r_{l}\left(1-c_{i2*}^{l}\right)\right)\;\;\right)\;\right). \end{array}$$

Finally, when $c_{i1*}^{k}=0$ for each *k*,

$$\begin{array}{c} L_{i}(\Theta |u_{i},v_{i})=\underset{k=h,f,l}{\prod }\left\{1-\exp\;\left(-u_{i}\omega_{i1}^{k}\right)\left\{1-\overset{k}{\underset{i1}{P}}+P_{i1}^{k}\left(1-P_{i2}^{k}\right)\exp\;\left(-v_{i}\omega_{i2}^{k}\right)\right\}\right\}\\ =\overset{1}{\underset{r_{h}=0}{\sum }}\overset{1}{\underset{r_{f}=0}{\sum }}\overset{1}{\underset{r_{l}=0}{\sum }}{\left(-1\right)}^{r_{h}+r_{f}+r_{l}}\exp\;\left(-u_{i}\underset{k=h,f,l}{\sum }r_{k}\omega_{i1}^{k}\right)\underset{k=h,f,l}{\prod }{\left\{1-\overset{k}{\underset{i1}{P}}+\overset{k}{\underset{i1}{P}}\left(1-\overset{k}{\underset{i2}{P}}\right)\exp\;\left(-v_{i}\overset{k}{\underset{i2}{\omega }}\right)\right\}}^{r_{k}}, \end{array}$$

where

$$\begin{array}{c} \underset{k=h,f,l}{\prod }{\left\{1-P_{i1}^{k}+P_{i1}^{k}\left(1-P_{i2}^{k}\right)\exp\;\left(-v_{i}\omega_{i2}^{k}\right)\right\}}^{r_{k}}\\ =\overset{r_{h}}{\underset{r_{h}^{\prime }=0}{\sum }}\overset{r_{f}}{\underset{r_{f}^{\prime }=0}{\sum }}\overset{r_{l}}{\underset{r_{l}^{\prime }=0}{\sum }}\underset{k=h,f,l}{\prod }{\left\{{\left[1-P_{i1}^{k}\right]}^{1-r_{k}^{\prime }}{\left[P_{i1}^{k}\left(1-P_{i2}^{k}\right)\right]}^{r_{k}^{\prime }}\right\}}^{r_{k}}\exp\;\left(-v_{i}\underset{k=h,f,l}{\sum }r_{k}^{\prime }\omega_{i2}^{k}\right). \end{array}$$

For each ${\left\{c_{i1*}^{k}\right\}}_{k=h,f,l}$ and ${\left\{c_{i2*}^{k}\right\}}_{k=h,f,l}$, it is now evident that the resulting likelihood contribution can be computed analytically if the distributional choice of *U*_*i*_ and *V*_*i*_ have closed form Laplace transforms, because *u*_*i*_ and *v*_*i*_ only appear as *a* exp(−*b* *u*_*i*_−*c* *v*_*i*_), where *a*,*b*,*c* are constants with respect to *u*_*i*_ and *v*_*i*_, for each likelihood contribution.

## References

[1] Aguirre‐Hernández R , Farewell VT . Appraisals of models for the study of disease progression in psoriatic arthritis In Advances in Survival Analysis, Balakrishnan N, Rao CR (eds), Vol. 23 Handbook of Statistics: Amsterdam 2004; 643–673.

[2] Cook RJ , Yi GY , Lee K , Gladman DD . A conditional Markov model for clustered progressive multistate processes under incomplete observation. Biometrics 2004; 60(2):436–443. 15180669 10.1111/j.0006-341X.2004.00188.x

[3] Solis‐Trapala IL , Farewell VT . Regression analysis of overdispersed correlated count data with subject specific covariates. Statistics in Medicine 2005; 24(16):2557–2575. 15977293 10.1002/sim.2121

[4] O'Keeffe AG , Tom BDM , Farewell VT . Mixture distributions in multi‐state modelling: some considerations in a study of psoriatic arthritis. Statistics in Medicine 2012; 32(4):600–619. 22833400 10.1002/sim.5529 PMC3575696

[5] Sutradhar R , Cook RJ . A bivariate mover‐stayer model for interval‐censored recurrent event data: application to joint damage in rheumatology. Communications in Statistics: Theory and Methods 2009; 38(18):3389–3405.

[6] Lambert D . Zero‐inflated Poisson regression, with an application to defects in manufacturing. Technometrics 1992; 34(1):1–14.

[7] Böhning D . Zero‐inflated Poisson models and C.A. Man: A tutorial collection of evidence. Biometrical Journal 1998; 40(7):833–843.

[8] Ridout M , Demtrio CGB , Hinde J . Models for count data with many zeros. *Proceedings of the XIXth International Biometrics Conference*, Cape Town, 1998 179–192.

[9] Böhning D , Dietz E , Schlattmann P . Zero‐inflated count models and their applications in public health and social science In Applications of Latent Trait and Latent Class Models in the Social Sciences. Waxmann: Munster, 1997.

[10] Hall DB . Zero‐inflated Poisson and binomial regression with random effects: A case study. Biometrics 2000; 56(4):1030–1039. 11129458 10.1111/j.0006-341x.2000.01030.x

[11] Hur K , Hedeker D , Henderson W , Khuri S , Daley J . Modeling clustered count data with excess zeros in health care outcomes research. Health Services and Outcomes Research Methodology 2002; 3(1):5–20.

[12] Lee AH , Wang K , Scott JA , Yau KKW , McLachlan GJ . Multi‐level zero‐inflated Poisson regression modelling of correlated count data with excess zeros. Statistical Methods in Medical Research 2006; 15(1):47–61. 16477948 10.1191/0962280206sm429oa

[13] Morgan CJ , Lenzenweger MF , Rubin DB , Levy DL . A hierarchical finite mixture model that accommodates zero‐inflated counts, non‐independence, and heterogeneity. Statistics in Medicine 2014; 33(13):2238–2250. 24443287 10.1002/sim.6091 PMC4057921

[14] Dobbie MJ , Welsh AH . Modelling correlated zero‐inflated count data. Australian and New Zealand Journal of Statistics 2001; 43(4):431–444.

[15] Conaway MR . A random effects model for binary data. Biometrics 1990; 46(2):317–328.

[16] Pregibon D . Goodness‐of‐link tests for generalized linear models. Journal of Applied Statistics 1980; 29(1):15–24.

[17] Broyden CG . The convergence of a class of double‐rank minimisation algorithms. Journal of the Institute of mathematics and its Applications 1970; 6(1):76–90.

[18] R Development Core Team . R: A Language and Environment for Statistical Computing. R Foundation for Statistical Computing: Vienna, Austria, 2008 http://www.R-project.org ,ISBN3-900051-07-0.

